# Small Molecule Inhibitor Adjuvant Surfactant Therapy Attenuates Ventilator- and Hyperoxia-Induced Lung Injury in Preterm Rabbits

**DOI:** 10.3389/fphys.2020.00266

**Published:** 2020-04-09

**Authors:** Pragnya Das, Tore Curstedt, Beamon Agarwal, Varsha M. Prahaladan, John Ramirez, Shreya Bhandari, Mansoor A. Syed, Fabrizio Salomone, Costanza Casiraghi, Nicola Pelizzi, Vineet Bhandari

**Affiliations:** ^1^Department of Pediatrics, Drexel University, Philadelphia, PA, United States; ^2^Department of Molecular Medicine and Surgery, Karolinska Institutet, Karolinska University Hospital, Stockholm, Sweden; ^3^GenomeRxUS, Secane, PA, United States; ^4^Department of Pediatrics, Yale University School of Medicine, New Haven, CT, United States; ^5^R&D Department, Chiesi Farmaceutici, Parma, Italy

**Keywords:** surfactant, mechanical ventilation, Ang2 siRNA, CHOP siRNA, miR34a inhibitor, preterm rabbit, neonates

## Abstract

**Background:**

Invasive mechanical ventilation (IMV) has become one of the mainstays of therapy in NICUs worldwide, as a result of which premature babies with extremely low birth weight have been able to survive. Although lifesaving, IMV can result in lung inflammation and injury. Surfactant therapy is considered a standard of care in preterm infants with immature lungs. Recently, small molecule inhibitors like siRNAs and miRNAs have been used for therapeutic purposes. Ddit3 (CHOP), Ang2 and miR34a are known to be upregulated in experimental lung injury. We wanted to test whether inhibitors for these molecules (CHOP siRNA, Ang2 siRNA, and miR34a antagomir) if used alone or with a combination with surfactant (Curosurf^®^) would help in reducing ventilation and hyperoxia-induced injury in an experimental lung injury model.

**Methods:**

Preterm rabbits born by cesarean section were intratracheally instilled with the three small molecule inhibitors with or without Curosurf^®^ prior to IMV and hyperoxia exposure. Prior to testing the inhibitors in rabbits, these small molecule inhibitors were transfected in mouse lung epithelial cells (MLE12 and AECII) and delivered to neonatal mouse pups intranasally as a proof of concept that surfactant (Curosurf^®^) could be used as an effective vehicle for administration of such drugs. Survival, pulmonary function tests, histopathology, immunostaining, quantitative PCR and western blotting were done to see the adjuvant effect of surfactant with these three small molecule inhibitors.

**Results:**

Our data shows that Curosurf^®^ can facilitate transfection of small molecules in MLE12 cells with the same and/or increased efficiency as Lipofectamine. Surfactant given alone or as an adjuvant with small molecule inhibitors increases survival, decreases IMV and hyperoxia-induced inflammation, improves pulmonary function and lung injury scores in preterm rabbit kits.

**Conclusion:**

Our study shows that Curosurf^®^ can be used successfully as an adjuvant therapy with small molecule inhibitors for CHOP/Ang2/miR34a. In this study, of the three inhibitors used, miR34a inhibitor seemed to be the most promising compound to combat IMV and hyperoxia-induced lung injury in preterm rabbits.

## Introduction

The preterm lung is highly susceptible to injury during resuscitation, and chronic invasive mechanical ventilation (IMV). Premature infants are most vulnerable to ventilator-induced lung injury (VILI) in the period immediately following birth because of their immature lungs with surfactant content that is often deficient and hence, not uniformly ventilated. IMV causes various degrees of lung injury that is characterized by endothelial and epithelial cell damage/death that activates an inflammatory response, all of which interferes with the pulmonary surfactant system (Bohlin et al., 2005). Infants with established respiratory distress syndrome (RDS) who receive animal-derived surfactant extract treatment have a decreased risk of pneumothorax, pulmonary interstitial emphysema and mortality (Seger and Soll, 2009). Thus, effective use of surfactant administration, reduces the risk of VILI (Keszler and Sant’anna, 2015).

Exogenous surfactant therapy alone has not been successful in decreasing this VILI (Bhat et al., 1990). Hence, with increasing knowledge of the detrimental effects of IMV and hyperoxia on the immature lung, there is a need for alternative treatment approaches to prevent hyperoxia-induced lung injury or VILI (Aldana-Aguirre et al., 2017). Recently, silencing/short interfering (si)RNAs and microRNAs (miRs) have been considered as potential therapeutic agents. So far, 20 clinical trials have been initiated using siRNAs and miRs (Chakraborty et al., 2017) and one drug, Patisiran (Onpattro) has been approved for use in the clinic (Garber, 2018). Hence, in the present study we wanted to explore the therapeutic potential of three small molecule inhibitors (CCAAT enhancer-binding protein, i.e., C/EBP homologous protein or CHOP siRNA, Angiopoietin 2 or Ang2 siRNA and miR34a antagomir/inhibitor or miR34a Inh) as candidates for treating ventilator and hyperoxia-induced lung injury in neonatal patients, born prematurely.

It has been reported that oxidative stress from prolonged hyperoxia leads to endoplasmic reticulum (ER) stress, resulting in activation of the unfolded protein response (UPR) and induction of CHOP, a transcription factor associated with cell death in the setting of persistent ER stress (Lozon et al., 2011). CHOP (also known as growth arrest and DNA damage-inducible gene 153/GADD153 or Ddit3) was selected based on our earlier work in which we reported that CHOP siRNA decreased cell death in alveolar epithelial cells and improved alveolarization in 2 different murine models of bronchopulmonary dysplasia (BPD) (Choo-Wing et al., 2013). Ang2 was chosen given our extensive characterization of its role in causing lung injury in experimental BPD murine neonatal models (Bhandari et al., 2006; Harijith et al., 2011; Sun et al., 2013b) as well as in human neonates (Aghai et al., 2008).

IMV and hyperoxia exposure induces changes in miRs (Vaporidi et al., 2012) which could precede measurable physiological/histological changes in the lungs. We have recently reported that miR34a levels are significantly increased in lungs of neonatal mice exposed to hyperoxia. Deletion or inhibition of miR34a improved the pulmonary phenotype and BPD-associated pulmonary arterial hypertension (PAH) in BPD mouse models, which, conversely, was worsened by miR34a overexpression (Syed et al., 2017). We had earlier reported that miR34a Inh, delivered using phosphate-buffered saline (PBS) as the vehicle to the airway (intranasally) reaches the target cells efficiently in the alveoli to attenuate the damage caused by hyperoxia exposure in the mouse model of BPD (Syed et al., 2017). Hence, we wished to test the hypothesis that CHOP siRNA, Ang2 siRNA and miR34a Inh delivered with exogenous surfactant poractant alfa (Curosurf^®^, Chiesi Farmaceutici, S.p.a., hereafter referred to as CS) as a vehicle into preterm rabbit lungs managed with IMV and supplemental oxygen, will result in attenuation of ventilator and hyperoxia-induced lung injury.

## Materials and Methods

### Captive Bubble Surfactometry

Captive bubble surfactometry was done following the methodology as reported in literature (Almlen et al., 2012). The test chamber was initially filled with 10% sucrose in saline. Approximately 2 μl of CS (5 mg/ml) was injected into the sample chamber and allowed to migrate by buoyance to the agarose ceiling. An air bubble was then placed under the ceiling in contact with CS and the pressure gradient across the bubble wall was recorded at 37°C during 50% cyclic surface compression at the rate of 40/min. Surface tension was assessed at maximal and minimal bubble sizes during the 5th cycle and after 1 and 5 min of pulsation surface tension was measured from the time of bubble insertion. The surface adsorption rate was then determined by arresting the pulsation at maximal bubble size and recording the time interval until static surface tension had dropped to the level of 30 mN/m. After 5 min of adsorption, the sample chamber was sealed and the quasi-static cycling was initiated. The bubble was compressed stepwise until a surface tension less than 5 mN/m was reached or to 50% area compression, and thereafter expanded to the initial size. This maneuver was repeated five times.

### Cell Culture

Mouse adult lung epithelial (adult-MLE12) and mouse fetal alveolar epithelial Type II cells (fetal-AECII) were transfected with 50 pmol of Ang2 siRNA, CHOP siRNA and miR34a Inh (Qiagen) either with Lipofectamine (LF) (3 μl; Invitrogen) or CS (5 μl; Chiesi). Three sets of the same experiment were conducted at different times to ensure reproducibility.

### Resuspension of CHOP siRNA, Ang2 siRNA, and miR34a Inh for Intratracheal Instillation, *in vivo*

The different siRNAs were dissolved in RNAse free water to make a stock solution of 600 nmol/ml (=600 μM). For the *in vivo* experiments 0.25 ml of siRNA was added to 0.75 ml of surfactant (Curosurf^®^ 80 mg/ml; Chiesi Farmaceutici, Parma, Italy) to get a final concentration of 60 mg/ml Curosurf^®^ (CS) and 150 nmol/ml of siRNA. A single dose of 500 nmol/kg siRNAs or miRNA Inh and 200 mg/kg Curosurf^®^ (CS) was administered to each animal, intratracheally. The preterm rabbits were randomized to one of the following groups for intratracheal instillation of the drugs:

1. Control animals (no treatment and sacrificed before the first breath).
2. Curosurf^®^ 200 mg/kg (60 mg/ml), 3.3 ml/kg
3. siRNA (150 nmol/ml) in Curosurf^®^ (60 mg/ml), 3.3 ml/kg
4. siRNA in RNAsefree water (150 nmol/ml), 3.3 ml/kg

## Animal Experiments

### Neonatal Mice

Wild type C57Bl/6 neonatal mouse pups were born in-house and exposed to 100% O_2_ following our standard protocols (Choo-Wing et al., 2007, 2013; Harijith et al., 2011; Sun et al., 2013a; Sureshbabu et al., 2016; Syed et al., 2017). Briefly, after the pups were born, the entire cage (mother + newborn pups) along with adequate food and water supply, were placed in the hyperoxia chamber (BioSpherix with ProOX Sensor 300, NY) connected to a continuous supply of oxygen (Airgas, NJ, United States) for four consecutive days. The newborn pups were allowed to be nursed with their mother, *ad libitum*, and the dams were cycled between litters exposed to room air and hyperoxia every 24 h to minimize acute oxygen toxicity for the mother and to ensure that the normoxic and hyperoxic dams received the same oxygen exposure. Soon after delivery (postnatal day 1, PN1), CHOP siRNA (20μM; 5 μl) with or without CS (12.5 mg/ml; 5 μl) was delivered intranasally on PN2 and PN4 during hyperoxia exposure following which they were sacrificed on PN5 and lungs harvested for further analysis. Only CHOP siRNA was chosen for the mice study, as a proof-of-concept.

### Preterm Rabbit Kits

For the rabbit experiments, preterm newborn rabbits (New Zealand White; *n* = 123) were obtained by cesarean section at a gestational age of 27–28 days (term, 31 days) following the methodology previously described (Berggren et al., 1985). As there were several technical challenges with handling the preterm rabbit kits, it was impossible to do all experiments at the same time. Hence, different kits were used from different cohorts at different times of the year.

For the controls (one set of animals with no treatment and the 2^*nd*^ set of animals receiving Curosurf^®^ only), a pilot experiment showed that it was difficult to keep preterm rabbits, especially those of 27 days gestational age (G27), alive and it was decided to use animals of 28 days gestational age (G28). Thus, both control animals (not given anything at all) and Curosurf^®^ treated preterm rabbits (G28) were ventilated for 4 h with 100% oxygen, at a constant tidal volume of 6–7 ml/kg and a positive end-expiratory pressure (PEEP) of 3 cm H_2_O (Supplementary Table S1).

For experiments following treatment with standardized pressure, G27 kits were used whereas for experiments following treatment with constant tidal volumes, G28 kits were used, for technical reasons. After delivery, the animals were randomized to different groups, as summarized in Table 1, anesthetized with an intraperitoneal injection of medetomidine (Domitor^®^), 0.1 mg/kg, and ketamine (Ketaminol^®^), 20 mg/kg, randomized for different treatments, tracheotomized and kept in plethysmograph boxes at 37°C. For the ventilation experiments, when the condition of the animals became stable after 120 min of Ketamine injection, they were administered with pentobarbital for a long-lasting effect of the anesthetic to withstand the changes in lung volume and tidal pressure. They were mechanically ventilated in parallel with a modified Servo-Ventilator (900B, Siemens-Elema, Solna, Sweden) with 100% oxygen, frequency of 40 breaths/min and an inspiration-to-expiration ratio of 1:1. Tidal volumes were adjusted using a PowerLab^®^ 4/20 system with Chart 5.2 software (ADInstruments Ltd., United Kingdom) equipped with a pneumotachometer (Hugo Sachs Elektronik-Harvard Apparatus, Germany) and a pressure transducer Capto SP 844 (Capto AS, Norway). Lung-thorax compliance was derived from recordings of tidal volume and peak inspiratory pressure and expressed as ml/kg cm H_2_O. This model has been previously shown to produce preterm rabbits with significant surfactant deficiency (i.e., RDS) that require supplemental oxygen and IMV for survival.

**TABLE 1:** Distribution of experimental groups.

Experimental group	Group name	Number of samples	Abbreviated as
Control (No CS – No ventilation)	CHOP siRNA	12	C w/o venti
Control (+ CS + ventilation)		12	CS
CHOP siRNA (+ ventilation)		8	CHOP siRNA
CHOP siRNA (+ CS + ventilation)		8	CHOP siRNA + CS
Control (No CS – No ventilation)	Ang2 siRNA	12	C w/o venti
Control (+ CS + ventilation)		12	CS
Ang2 siRNA (+ ventilation)		10	Ang2 siRNA
Ang2 siRNA (+ CS + ventilation)		10	CHOP siRNA + CS
Control (No CS – No ventilation)	miR34a Inh	8	C w/o venti
Control (+ ventilation)		8	C w venti
Control (+ CS + ventilation)		12	CS
miR34a inhibitor (+ ventilation)		5	miR34a Inh
miR34a inhibitor (+ CS + ventilation)		6	miR34a Inh + CS

For each experiment with the specific siRNAs/miR-antagomir, a CS group (control) was used. The experiments were performed at different times of the year depending on the availability of pregnant females. They were done by the same experienced team, at the same research laboratory using the same protocols.

The animal protocols were approved by Drexel University, United States for the mice study and Stockholms Norra Djurförsöksetiska Nämnd N174/14 for the rabbit study.

### Pulmonary Functions Tests (PFTs) in Rabbit Kits

#### IMV With Standardized Pressures

A screening method with standardized pressures was performed to evaluate the effect of a small molecule inhibitor on surfactant activity in preterm rabbits. The newborn rabbits (G27) were ventilated with 100% oxygen in parallel with a standardized sequence of peak insufflation pressures and an inspiration: expiration ratio 1:1. To open up the lungs, pressure was first set at 35 cm H_2_O for 1 min. After this recruitment maneuver, pressure was lowered to 25 cm H_2_O for 15 min and further on to 20 and 15 cm H_2_O. Finally, pressure was raised again to 25 cm H_2_O for 5 min after which the lungs were ventilated for additional 5 min with nitrogen to stabilize the lung gas volumes. The tracheal cannula was clamped at end expiration, the trachea was ligated and the lungs were excised and weighed. Lung gas volumes were determined by water displacement technique (Scherle, 1970). The experiments were performed without PEEP.

Pilot experiments were performed on captive bubble surfactometry and on extremely premature rabbits (G27 only) on CHOP siRNA to assess if small molecule inhibitors had any impact on the surface activity of CS. The results show that the mixture of CS and CHOP siRNA was equally surface active, as CS alone, *in vitro* (Supplementary Figure S1 and Table 2).

**TABLE 2:** Minimum (γ_min_) and maximum (γ_max_) surface tension as well as percent compression (area%) to reach a surface tension of 5 mN/m were evaluated for the 5th cycle using the captive bubble surfactometer.

Surfactant	Concentration	No. of experiments	γ_min_ (mN/m)	γ_max_ (mN/m)	% compression (to obtain 5 mN/m)
CS	5 mg/ml	3	0.8 ± 0.1	26.4 ± 1.3	14.6 ± 1.6
CHOP siRNA	150 nmol/ml	3	>25	>50	>50
CS + CHOP siRNA	5 mg/ml + 150 nmol/ml	5	1.1 ± 0.2	27.2 ± 1.5	17.3 ± 1.6

*The different surfactant suspensions were measured 3–5 times and the values are given as mean ± SD.*

#### IMV With Constant Tidal Volume

The newborn kits, usually at G28 (except some non-ventilated control animals at G27), were connected to ECG and ventilated in parallel with individual pressures in order to keep standardized tidal volumes. To inflate the lungs, pressure was first set at 35 cm H_2_O for 1 min. After this recruitment maneuver, pressure was lowered to keep tidal volumes at 6–7 ml/kg but with the limitation that the maximum pressure applied was 25 cm H_2_O. The animals were ventilated with 100% oxygen, inspiration: expiration ratio 1:1 and a PEEP of 3cm H_2_O. After 120 min the animals were given 10 ml/kg glucose and pentobarbital 24 mg/kg. Dead animals as well as those with an ECG less than 60/min were excluded from the study. After 4 h the animals were euthanized, the diaphragms were inspected for evidence of pneumothorax and the lungs were excised. Both tidal volumes and lung gas volumes were determined.

##### Histology

The left lungs were consistently used for histology. After the kits were euthanized, the lungs were harvested and fixed in 4% paraformaldehyde for Hematoxylin-Eosin staining and TUNEL staining.

##### Immunostaining

Fluorescent immunostaining was done for von Willebrand factor (vWF, 1:100, DAKO) as previously described (Leary et al., 2019).

##### TUNEL staining

TUNEL staining was done using the TUNEL kit (Roche) following the manufacturer’s instructions.

##### Real time PCR

Quantitative PCR (qPCR) was done to assess changes in cytokine levels in lung tissue, as previously described (Leary et al., 2019). The primers used are shown in Supplementary Table S2, with sequences.

##### Western blotting

Immunoblotting was done as previously described (Leary et al., 2019). The antibodies used were Ang1, Ang2 (Sigma; 1:500), β-actin (1:2000), interleukin-1beta or IL1β (1:50), IL6 (MyBioSource; 1:50), CHOP (Cell Signaling; 1:1000) and Sirt1 (Lifespan BioSciences; 1:500). Briefly, 30 μg of protein lysates were run on 4–20% SDS PAGE-gels (Genscript, United States), immunoblotted with the above antibodies, followed by incubation with fluorescent conjugated appropriate secondary antibodies (Li-COR, 1:10,000) and developed using infrared imager (Li-COR).

##### Lung injury scoring

Lung histopathology was assessed, and scoring was done following the method as described (Matute-Bello et al., 2011) with slight modifications, by a qualified blinded pathologist. Slides were scored in high power (40X) fields and lung injury was categorized based on the presence of neutrophils in alveolar space, neutrophils in interstitial space, interstitial congestion and thickening, presence of hyaline membranes, presence of proteinaceous debris and overall alveolar morphology. Each category of injury was graded with a score from 0 to 5 (0 – no injury to 5 – highest injury). The final scores were given based on the formula as cited by Matute-Bello et al. (2011).

##### Statistics

All statistical analyses were performed using Graph Pad Prism, version 8.0 (GraphPad Software, San Diego, CA, United States). The data are expressed as the mean ± SEM (or SD) with *n* = 8–12 kits for survival/PFTs in each group, *n* = 10 for qPCR analysis, *n* = 6–8 for lung histology/lung injury scoring/Western Blotting. Survival was analyzed using Kaplan Meier plots. Cell cultures were done in triplicates. Groups were compared with the Student’s two-tailed unpaired *t*-test and one-way or two-way ANOVA, as appropriate, followed by Tukey *post hoc* analysis. A *p* ≤ 0.05 was considered statistically significant.

## Results

### CS as a Promising Delivery Vehicle for Small Molecule Inhibitors, *in vitro* and *in vivo*

A pilot experiment on captive bubble surfactometry was performed to assess if small molecule inhibitors had any impact on the surface activity of CS. The results show that the mixture of CS and CHOP siRNA was equally surface active as CS alone, *in vitro* (Table 2).

**FIGURE 1:**
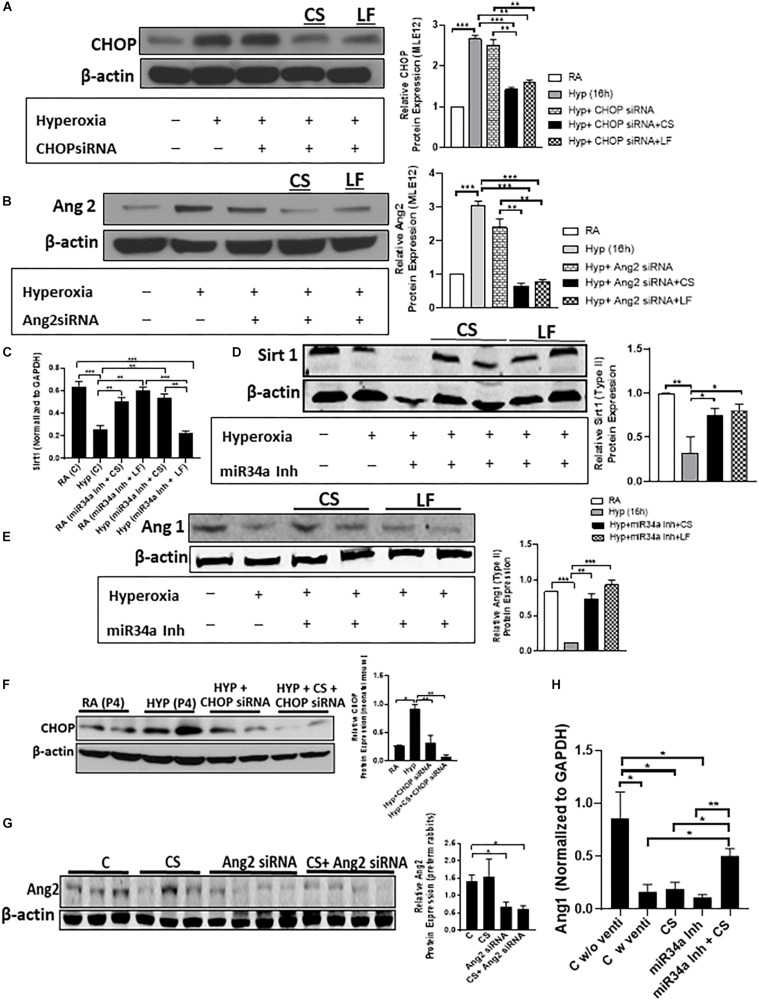
CS can be used as a successful delivery vehicle, *in vitro* and *in vivo*. Adult epithelial MLE12 cells **(A,B)** and fetal type II alveolar epithelial cells **(C–E)** were transfected with CHOP siRNA, Ang2 siRNA, and miR34a Inh, respectively, following which the expression of CHOP **(A)**, Ang2 **(B)**, Sirt 1 **(D)**, and Ang1 **(E)** (Sirt1 and Ang2 are targets of miR34a) were quantified by Western Blotting **(A,B,D,E)** with respective densitometric analysis (extreme right) and qPCR **(C)**. Lipofectamine (LF) was used as a positive control. The cells that were treated with Curosurf^®^ (CS), showed equal efficiency of transfection as that of LF, as is evident from decreased expression of CHOP and Ang2. miR34a is increased during hyperoxia, but after treatment with CS, the expression of miR34a is suppressed which leads to an increase in Sirt1 **(C,D)** or Ang1 **(E)** expression. Neonatal mouse pups were intranasally delivered with CS + CHOP siRNA in each nostril on PN2 and PN4 of hyperoxia treatment. Western blotting shows decreased expression of CHOP **(F)**, along with densitometric quantification (on the right). Ang2 siRNA was administered intratracheally along with CS in preterm rabbit kits. Western blotting shows decreased expression of Ang2 **(G)**, along with densitometric quantification (on the right). Q-PCR showing relative expression of Ang1 after treatment with miR34a inh **(H)**. On treatment with miR34a Inh alone, we saw a significant decrease in the expression of Ang1 (target of miR34a) in the ventilated group as compared to non-ventilated controls. However, adjuvant therapy with a combination of CS + miR34a Inh restored the level of Ang1 above the basal level, which was otherwise compromised after ventilation **(H)**. β-actin is the loading control for all the western blotting while GAPDH was used as a normalizing gene for qPCR. ^∗^*p* ≤ 0.05; ^∗∗^*p* ≤ 0.001; ^∗∗∗^*p* ≤ 0.0001; (*N* = 2–4). CS: Curosurf^®^ (surfactant); CHOP: CCAAT enhancer-binding protein, i.e., C/EBP homologous protein, also known as growth arrest and DNA damage-inducible gene 153/GADD153 or Ddit3; si: silencing; Ang2: Angiopoietin 2; miR: microRNA; Inh: inhibitor; Sirt: Sirtuin; LF: Lipofectamine; RA: Room Air. Hyp: Hyperoxia. Overall, the data convincingly prove that CS can be used as an effective vehicle for administration of test agents; CS is stable when used in combination with the small molecule inhibitors, and the latter are capable of successfully suppressing the expression of the proteins of interest, following injury.

MLE12 and AECII cells were transfected with 50 pmol CHOP siRNA, or Ang2 siRNA or miR34a Inh and CS (5 μl) and LF (3 μl), followed by hyperoxia treatment for 16 h (Figures 1A–E). CHOP and Ang2 expression decreased following transfection with the respective siRNAs (Figures 1A,B). We checked the expression of Ang1 and Sirt1, *in vitro* [the two confirmed target proteins of miR34a as has been previously described by us (Syed et al., 2017)] by qPCR (for Sirt1, Figure 1C). Ang1 and Sirt1 were decreased after hyperoxia treatment, but after addition of miR34a Inh and CS or LF, both the target proteins were upregulated (Figures 1D,E). Neonatal mice pups were administered CHOP siRNA + CS intranasally on PN2 and PN4 following hyperoxia exposure while G28 preterm rabbit pups were intra-tracheally instilled with Ang2 siRNA + CS or miR34a Inh + CS. CS was able to deliver the compounds effectively as evident from the decreased expression of CHOP (Figure 1F) and Ang2 (Figure 1G). Ang1 was significantly increased in the group treated with miR34a Inh + CS than with CS alone or miR34a Inh alone (Figure 1H). Further, addition of miRNA Inh to CS did not have any negative effect on tidal volume (Supplementary Figure S1A), compliance (Supplementary Figure S1B) and lung gas volume (Supplementary Figure S1C) in experiments with standardized pressures, in preterm rabbits. All the three small molecule inhibitors could not be tested at the same time due to technical reasons.

### Developmental Regulation and Impact of Ventilation With Hyperoxia on CHOP, Ang2, and miR34a Expression in the Preterm Rabbit Model

**FIGURE 2:**
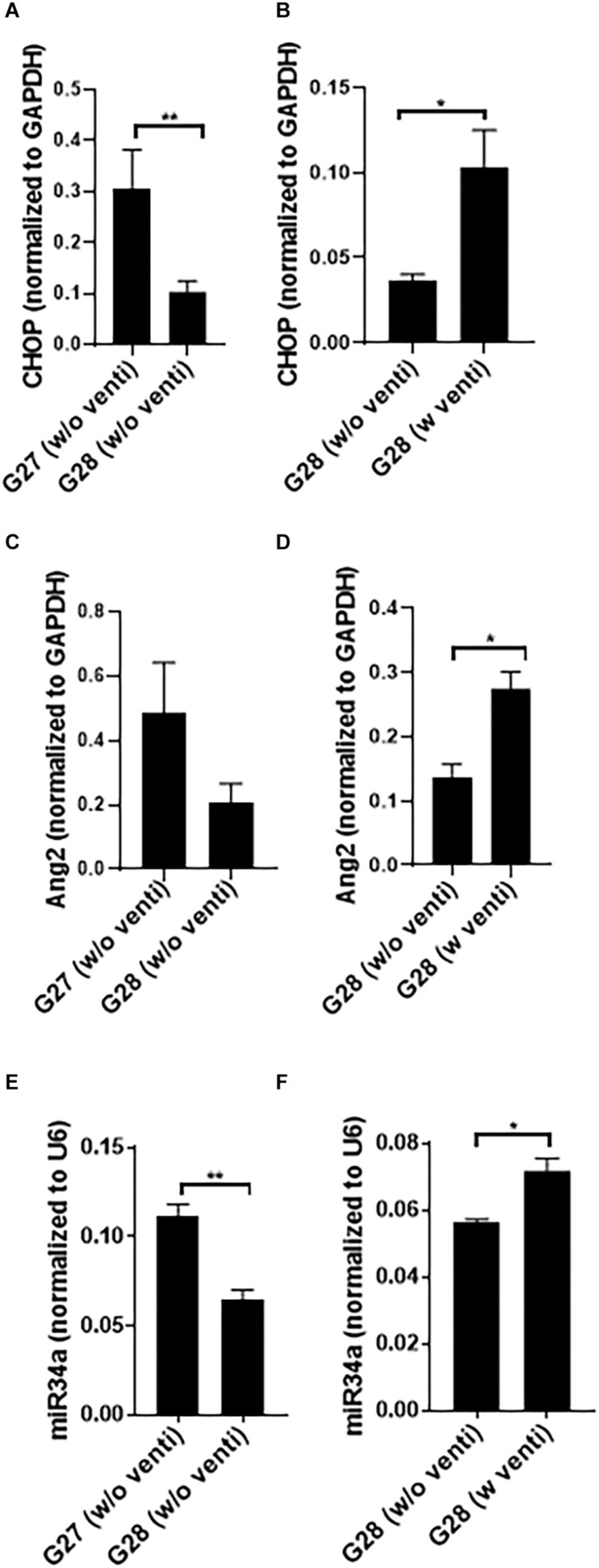
Developmental regulation and impact of ventilation with hyperoxia on CHOP, Ang2 and miR34a expression in the preterm rabbit model. Quantitative PCR showing a comparative expression of CHOP **(A)**, Ang2 **(C),** and miR34a **(E)** at G27 and G28 with no ventilation support. At G27, CHOP, and miR34a increase significantly as compared to G28 **(A,E)** while there was no change in the expression of Ang2 **(C).** However, at G28, CHOP, Ang2 as well as miR34a increase significantly with ventilation with hyperoxia compared to non-ventilated controls **(B,D,F)**. The G28 groups were done at different times; hence they have been shown in different graphs ^∗^*p* ≤ 0.05; ^∗∗^*p* ≤ 0.001; *N* = 6–8, per group. CHOP: CCAAT enhancer-binding protein, i.e., C/EBP homologous protein, also known as growth arrest and DNA damage-inducible gene 153/GADD153 or Ddit3; Ang2: Angiopoietin 2; miR: microRNA; G: gestational age (day).

The expression of CHOP and miR34a was significantly higher at G27 as compared to G28 (Figures 2A,E) with no change in Ang2 (Figure 2C). miR34a expression was maximum at 240 min of ventilation as compared to 300 min at G28 (data not shown). The kits did not survive beyond 300 min post ventilation; with increased mortality at G27, compared to G28. CHOP, Ang2 and miR34a were significantly increased in the ventilated, compared to the non-ventilated, group (Figures 2B,D,F). Hence, studies were conducted at G28 with ventilation support for 240 min.

### CS Can Prolong Survival as an Adjuvant With Small Molecule Inhibitors

**FIGURE 3:**
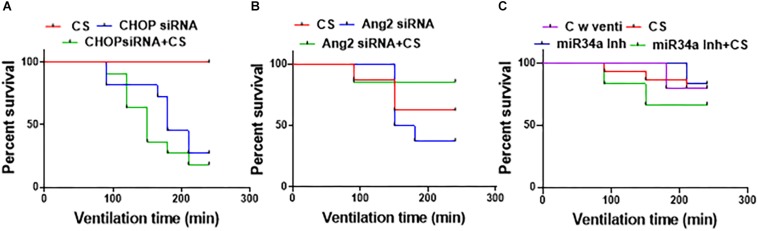
Effect of surfactant with small molecule inhibitors on survival. All the groups were ventilated for 240 min (*N* = 6–8, per group). Treatment with CHOP siRNA or CHOP siRNA + CS. **(A)** CS alone did not cause any deaths in the groups treated with CHOP; however, there was a significant decline in the survival with CHOP treatment alone or with a combination of CHOP + CS (*p* = 0.0003); 33.3% survived in the CHOP group, 20% in the CHOP + CS and 100% in the CS group **(A)**. Treatment with Ang2 siRNA or Ang2 siRNA + CS **(B)**: The survival was 62% for the group with CS alone, 62.5% for Ang2 and 85.7% for Ang2 + CS; Treatment with miR34a Inh or miR34a Inh + CS. **(C)** Of all the groups treated with a combination of CS, the ones treated with miR34a Inh + CS survived the best till 240 min with an overall survival of 75%. There was 70% survival for miR34a Inh + CS, 83% for miR34a Inh, 85.7% for CS and 72.7% for control with ventilation only; not statistically different. CHOP: CCAAT enhancer-binding protein, i.e., C/EBP homologous protein, also known as growth arrest and DNA damage-inducible gene 153/GADD153 or Ddit3; CS: Curosurf^®^ (surfactant); Ang2: Angiopoietin 2; si: silencing; miR: microRNA; Inh: inhibitor; min: minutes.

Of all the groups adjuvantly treated with a combination of CS (Figures 3A–C), the ones treated with miR34a Inh + CS survived the best (till 240 min) with an overall survival of 75%. There was a significant decline in the survival with CHOP siRNA treatment alone or with a combination of CHOP + CS (*p* = 0.0003); 33.3% survived in the CHOP group, 20% in the CHOP + CS group and 100% in the CS group. There was no significant difference in the survival of groups treated with Ang2 siRNA or miR34a Inh, either alone or in combination with CS versus CS alone. There was 72.7% survival for the control group with ventilation, 85.7% for CS alone, 83% for miR34a Inh and 70% survival for miR34a Inh + CS. The survival was 62% for the group with CS, 62.5% for Ang2 siRNA and 85.7% for Ang2 siRNA + CS. Of all the 3 groups of treatment, the miR34a Inh alone (Figure 3C) and Ang2 siRNA with CS (Figure 3A) had the highest rates of survival. Many of the animals were excluded from the study because they could not survive, especially those treated with CHOP siRNA with and without CS (Supplementary Table S3).

### PFTs After Adjuvant Treatment of CS With Small Molecule Inhibitors

**FIGURE 4:**
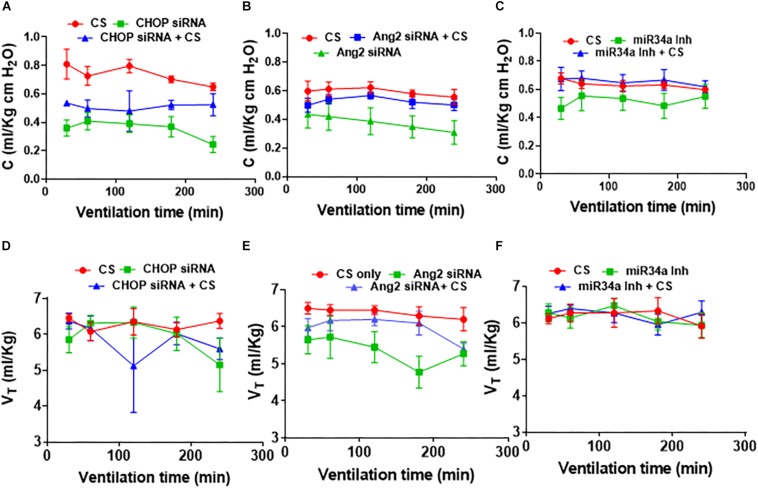
Effect of CS treatment with small molecule inhibitors on pulmonary function. In CHOP **(A)** or Ang2 **(B)** siRNA with/without CS treated groups, CS treatment alone was the most compliant (*p* < 0.001 at 60 through 240 min for CHOP siRNA; *p* = 0.02 at 180 and 240 min for Ang2 siRNA). In the Ang2 group, treatment with a combination of Ang2 siRNA + CS resulted in decreased lung compliance (*p* = 0.07) **(B)**, while in the CHOP group, treatment with CHOP siRNA + CS resulted in increased lung compliance than treatment with CHOP siRNA alone (*p* = 0.05) **(A)**. miR34a Inh showed the best compliance with no significant difference in all the three groups (CS with ventilation, miR34a Inh with ventilation and miR34a Inh + CS with ventilation) studied **(C)**. In the CHOP group, no difference was observed at the different time points studied, except at 240 min when CS treatment alone showed better tidal volume than the rest of the groups (*p* = 0.03) **(D)**. In the Ang2 group, CS alone was able to maintain the tidal volume better than when treated with a combination of Ang2 siRNA or Ang2 siRNA alone at a time point of 120 min through 240 min (*p* = 0.01) **(E)**. The overall tidal volume through all time periods was significantly less (*p* = 0.005) with Ang2 siRNA treatment than when treated with CS alone **(B)**. In the miR34a group, no statistical difference in the lung volume was noted at all time points starting from 30 min through 240 min of ventilation **(F)**. CS: Curosurf^®^ (surfactant); Ang2: Angiopoietin 2; si: silencing; min: minutes; CHOP: CCAAT enhancer-binding protein, i.e., C/EBP homologous protein, also known as growth arrest and DNA damage-inducible gene 153/GADD153 or Ddit3; miR: microRNA; Inh: inhibitor.

In CHOP (Figure 4A) or Ang2 (Figure 4B) siRNA with/without CS treated groups, CS treatment alone was the most compliant (*p* < 0.001 at 60 through 240 min for CHOP siRNA; *p* = 0.02 at 180 and 240 min for Ang2 siRNA). In the Ang2 group, treatment with a combination of Ang2 siRNA + CS showed a trend toward decreased lung compliance (*p* = 0.07) (Figure 4B) while in the CHOP group, treatment with CHOP siRNA + CS resulted in increased lung compliance than treatment with CHOP siRNA alone (*p* = 0.05) (Figure 4A). miR34a Inh showed the best compliance with no significant difference in all the three groups (CS with ventilation, miR34a Inh with ventilation and miR34a Inh + CS with ventilation) studied (Figure 4C). In summary, there was no statistically significant difference in the lung compliance measurements in the three experimental groups tested. miR34a Inh along with CS, showed a trend toward being more compliant, as compared with other groups (Figure 4C). A linear regression analysis showed that in the Ang2 group, CS alone was able to maintain the tidal volume better than when treated with a combination of Ang2 siRNA + CS or Ang2 siRNA alone, at a time point of 120 min through 240 min (*p* = 0.01). The overall tidal volume through all time periods was significantly less (*p* = 0.005) with Ang2 siRNA treatment than when treated with CS alone (Figures 4D–F).

### CS as an Adjuvant Approach for Suppressing Cytokine Induced Inflammation During IMV and Hyperoxia Exposure

**FIGURE 5:**
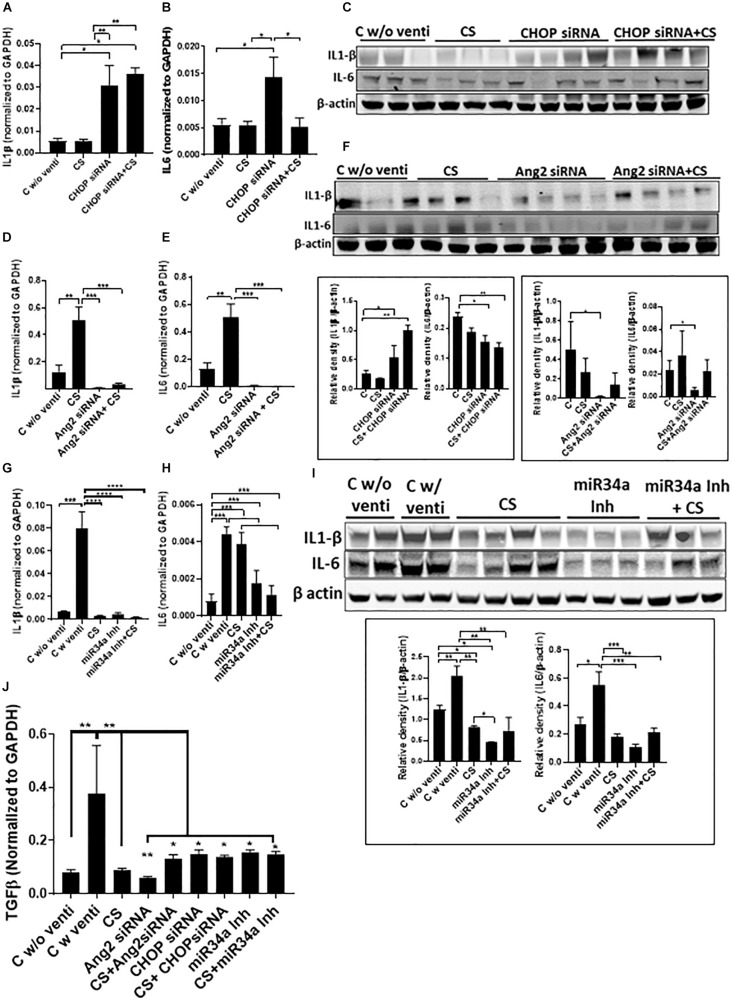
Adjuvant effect of CS and small molecule inhibitors on inflammation. Expression of IL1β and IL6 by Q-PCR and western blot analysis after treatment with CHOP siRNA **(A–C)** or Ang2 siRNA **(D–F)** or miR34a Inh **(G–I)**. CHOP siRNA alone significantly increased IL1β and IL6 mRNA; however, in combination with CS, IL1β remained increased, while IL-6 mRNA was significantly decreased, compared to CHOP siRNA alone (and similar to non-ventilated or CS alone treated controls) **(A,B)**. IL1β protein was similarly increased, while IL6 was significantly decreased **(C)**. There was a significant decrease in IL1β and IL6 mRNA with Ang2 siRNA alone or in combination with CS, compared to controls **(D,E)**. Both IL1β and IL6 proteins were significantly decreased with Ang2 siRNA alone compared to non-ventilated controls **(F)**. There was a significant decrease in IL1β mRNA with CS alone, miR34a Inh alone or with miR34a Inh + CS, compared to ventilated controls **(G)**. There was a significant decrease in IL6 mRNA with miR34a Inh alone or with miR34a Inh + CS, compared to ventilated controls alone or with CS **(H)**. There was a significant decrease in IL1β and IL6 proteins with CS alone, miR34a Inh alone or with miR34a Inh + CS, compared to ventilated controls **(I)**. There was a significant increase in TGFβ expression with ventilation in the controls but the expression decreased with CS alone as well the 3 small molecule inhibitors alone or with CS **(J)**. ^∗^*p* ≤ 0.05; ^∗∗^*p* ≤ 0.001; ^∗∗∗^*p* ≤ 0.0001; (*N* = 6–8). CS: Curosurf^®^ (surfactant); IL: interleukin; CHOP: CCAAT enhancer-binding protein, i.e., C/EBP homologous protein, also known as growth arrest and DNA damage-inducible gene 153/GADD153 or Ddit3; si: silencing; Ang2: Angiopoietin 2; miR: microRNA; Inh: inhibitor; TGFβ: transforming growth factor beta.

IL1β increased significantly when treated with CHOP siRNA with or without CS as compared to CS alone (Figures 5A,C). IL6 was increased significantly after treatment with CHOP siRNA but showed a significant decrease when used in combination with CS + CHOP siRNA, comparable to the level as seen in normal controls and with CS treated group alone (Figures 5B,C). In the Ang2 siRNA treated group, both IL1β and IL6 were high in the CS treated group but the levels significantly decreased after treatment with either Ang2 siRNA or Ang2 siRNA + CS (Figures 5D–F). Similarly, in the miR34a Inh group, there was a significant increase in IL1β and IL6 in the control group, ventilated soon after birth. However, with CS, the expression of IL1β, but not IL6, decreased significantly. Interestingly, miR34a Inh alone or a combination of CS + miR34a Inh led to a significant decrease of both IL1β and IL6 (Figures 5G–I). Transforming growth factor beta (TGFβ) expression was predominantly threefold higher than the groups that were not ventilated or ventilated with CS alone or with a combination of CS and the three small molecule inhibitors used in this study (Figure 5J).

### Effect of Delivery of Small Molecule Inhibitors With or Without CS on Lung Injury

**FIGURE 6:**
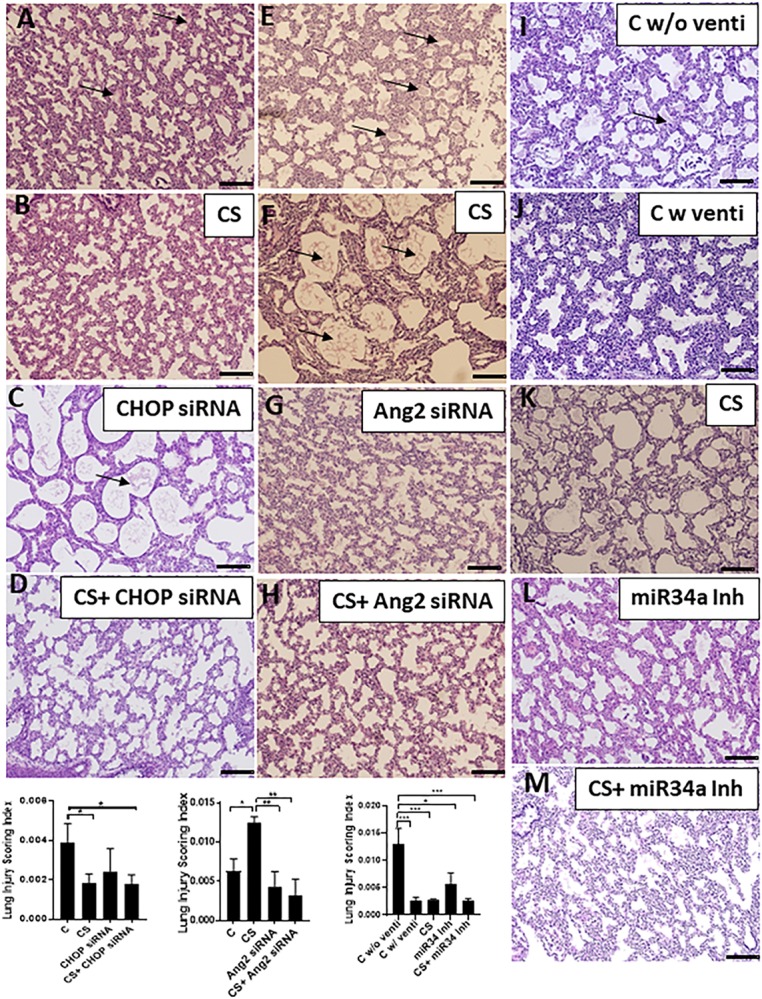
Adjuvant effect of CS and small molecule inhibitors on histopathology. In the CHOP siRNA group **(A–D)**, the controls were unventilated **(A)**, and showed interstitial thickening between the pneumocytes with hyaline membrane deposits in the alveolar spaces. Although CS treatment helped in the recovery of lung injury in the CHOP siRNA group **(B)**, treatment with CHOP siRNA alone **(C)** did not show any significant improvement, but there was significant improvement in the CHOP siRNA + CS group **(D)** as shown in the Lung Injury Scoring Index graph. In the Ang2 siRNA group **(E–H)**, the controls were unventilated **(E)**, and showed interstitial thickening between the pneumocytes with hyaline membrane deposits in the alveolar spaces. CS treatment did not help in the recovery of lung injury in the **(F)**, treatment with Ang2 siRNA alone **(G)** or in combination with CS **(H)** showed significant improvement, as shown in the Lung Injury Scoring Index graph. In the miR34a Inh group **(I–M)**, the controls were unventilated **(I)**, or ventilated **(J)** with the latter improvement. CS treatment alone significantly helped in the recovery of lung injury **(K)**, which was also noted in the animals treated with miR34a Inh alone **(L)** or in combination with CS **(M)**, as shown in the Lung Injury Scoring Index graph. Arrows represent hyaline membrane deposits. Scale bar: 200μm; ^∗^*p* ≤ 0.05; ^∗∗^*p* ≤ 0.001; ^∗∗∗^*p* ≤ 0.0001; (*N* = 6–8). CS: Curosurf^®^ (surfactant); CHOP: CCAAT enhancer-binding protein, i.e., C/EBP homologous protein, also known as growth arrest and DNA damage-inducible gene 153/GADD153 or Ddit3; si: silencing; Ang2: Angiopoietin 2; miR: microRNA; Inh: inhibitor.

While comparing the histopathology of all the groups, we found that lungs from the control group (born prematurely with no ventilation) were atelectatic with increased hyaline membrane deposits (Figures 6A,E,I). CHOP siRNA alone or in combination with CS had significantly (^∗^*p* < 0.05) decreased lung injury scores, compared to unventilated controls (Figures 6A,C,D). Ang2 siRNA alone or in combination with CS had significantly (^∗^*p* < 0.05, ^∗∗^*p* < 0.01) decreased lung injury scores, compared to unventilated as well as CS ventilated controls (Figures 6E–H). miR34a Inh alone or miR34a Inh + CS had significantly decreased lung injury scores (^∗^*p* < 0.05), compared to ventilated alone controls or other experimental groups (Figures 6I–M).

### CS With Small Molecule Inhibitors Can Attenuate Vascular Injury Following IMV

**FIGURE 7:**
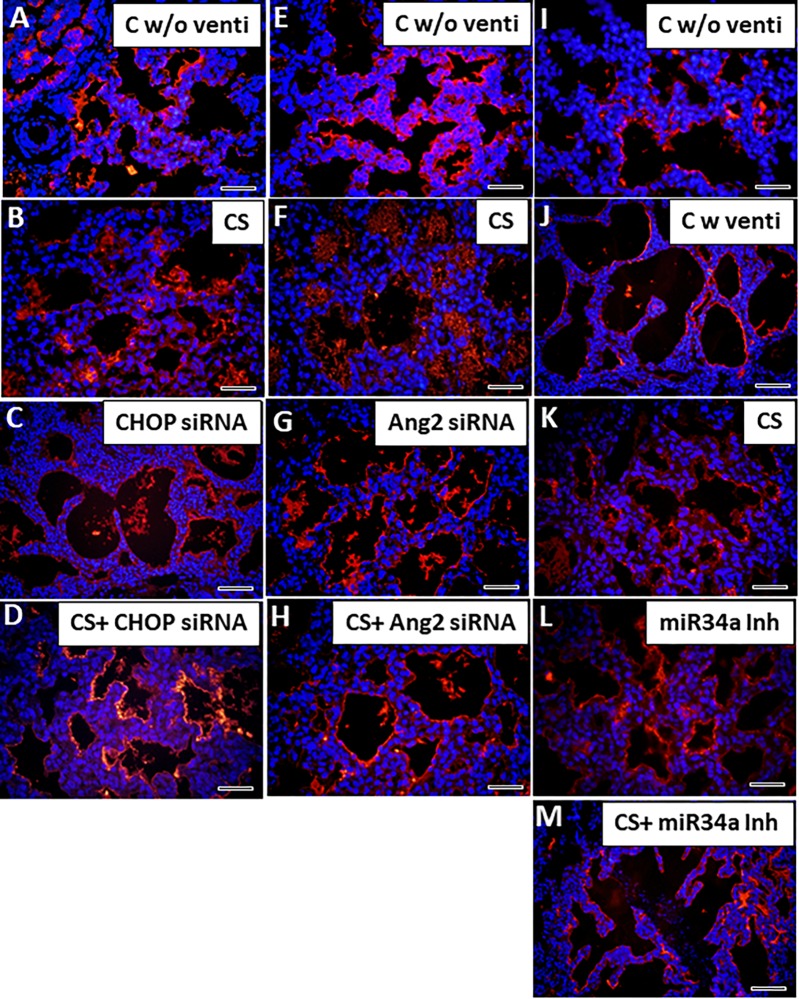
Adjuvant effect of CS and small molecule inhibitors on angiogenesis. Representative immunofluorescent staining with vWF showing vascular patterning with or without CS. Immediately after birth following a c-section (without ventilation) the blood vessels are thin, look fragile and are less in number **(A,E,I)**. Following ventilation, the alveoli are expanded leading to normal looking uniform and continuous vessels **(J)**. On treatment with CS (which is accompanied by IMV), the vessels are irregular, but more in numbers **(B,F,K)** as compared to the ones without ventilation. Treatment with CHOP siRNA or Ang2 siRNA or miR34a Inh individually or with CS, restored the normal vascular patterning **(C,D,G,H,L,M)**. Only in the miR34a group, there were unventilated controls. Scale bar: 100 μm.

IMV is associated with impaired vascular growth resulting in alterations in pulmonary vascular remodeling and tone, thus increasing the risk for persistent hypoxemia associated with pulmonary hypertensions in neonates who develop BPD (Alvira, 2016). In the present study, premature birth led to atelectasis in lungs characterized by sparsely distributed scant blood vessels which were largely dysmorphic (Figures 7A,E,I). After ventilation, as the alveoli opened up, the blood vessels were dilated and appeared more organized probably due to an increase in pulmonary blood flow (Figure 7J), and damaged due to the mechanical force of ventilation, as was evident from discontinuous staining by vWF (marker for endothelial cells). These vessels were also thickened (Figures 7B–D,F–H) following ventilation as compared to unventilated controls. However, after treatment with CS accompanied with IMV, the physical damage to the vessels was probably restored; there was no disruption in the expression of vWF, but there was accumulation of remnant CS in the empty alveolar spaces (Figures 7C,G). Treatment with either CS alone or with CHOP siRNA or Ang2 siRNA or miR34a Inh (Figure 7L) individually, restored the normal vasculature patterning as is usually expected in full term controls; adjuvant treatment of CS with the three small molecule inhibitors further improved vascular remodeling including those of pulmonary arteries (Supplementary Figure S2A–H) and decreased accumulation of residual CS in the alveolar spaces, which was not observed when treated with CS alone. Intense vWF staining was observed in all the ventilated groups treated with or without CS. Of the 3 groups, CS + miR34a Inh showed the best response to recovery of the vascular pattern as was characterized by thin, continuous, normal looking vessels lining the endothelium around the alveolar epithelium (Figure 7M).

### CS Can Potentially Decrease Cell Death Following IMV and Hyperoxia

**FIGURE 8:**
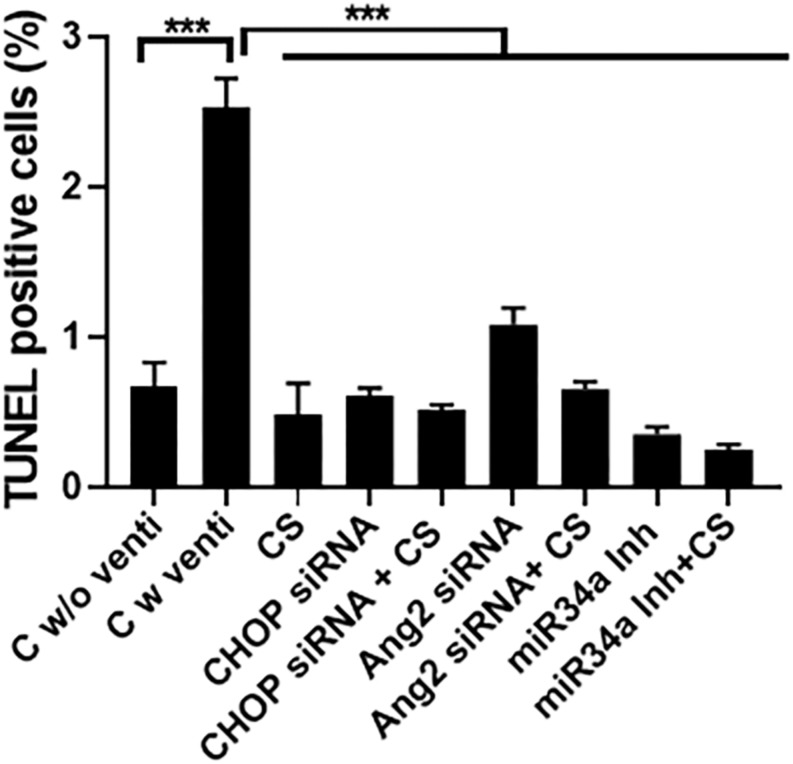
Effect of CS and small molecule inhibitors on cell death. Quantification of TUNEL staining in all the experimental groups treated with or without CS, either alone or as an adjuvant with the three small molecule inhibitors. There was maximal cell death in the control ventilated group, as compared to the others. Upon treatment with CS alone or in combination with CHOP siRNA, Ang2 siRNA or miR34a Inh there was a significant decrease in TUNEL positive cells. ^∗∗∗^*p* ≤ 0.0001; (*N* = 8–10). CS: Curosurf^®^ (surfactant); CHOP: CCAAT enhancer-binding protein, i.e., C/EBP homologous protein, also known as growth arrest and DNA damage-inducible gene 153/GADD153 or Ddit3; si: silencing; Ang2: Angiopoietin 2; miR: microRNA; Inh: inhibitor.

There was increased cell death in the control ventilated group as compared to either CS or the three small molecule inhibitors alone or with a combination of CS + CHOP siRNA or CS + Ang2 siRNA or CS + miR34a Inh (Figure 8), as was evident from TUNEL staining.

## Discussion

Prematurity remains the common denominator in the heterogeneous spectrum of VILI and preterm animal models have a clear translational advantage as modeling systems to understand the pathogenesis associated with VILI. The preterm rabbit model is a promising model as it includes a factor of prematurity with exposure to IMV and supplemental O_2_ (hyperoxia). They are phylogenetically related to and have an anatomy and physiology that more closely resembles that of primates. In many instances this leads to a more accurate modeling of human conditions. Based on these facts, we chose preterm rabbits, born at G28 by c-section, as our experimental model, for this study.

The decreased surfactant pool found in preterm lung can contribute to non-uniform expansion of the lung leading to atelectasis (Jobe et al., 2008). A short period of IMV can cause a decrease in lung compliance that is associated with a large influx of proteins into the alveolar space resulting in alterations of the pulmonary surfactant system (Veldhuizen et al., 2000). To test the fact that CS can be used to compensate for this loss, we used CS as a delivery vehicle as well as an adjuvant to suppress injury associated with IMV and hyperoxia. We first tested CS on survival alone, of the preterm rabbits, to ensure that it could effectively decrease mortality. In the CHOP siRNA treated group, CS showed the most promising result with 100% survival as compared to the other 2 groups. A combination of CS + Ang2 siRNA increased survival while miR34a Inh alone was sufficient for survival of the preterm kits. Similarly, CS was also able to increase the pulmonary function either alone or in combination with the other test agents. This proves that CS can be used as an effective adjuvant therapy. In the case of therapeutic agents such as miR34a Inh where the compound itself was able to improve survival, CS did not have an additive effect on the survival response, but in the scenario of CHOP siRNA or Ang2 siRNA treated groups, addition of CS further improved survival, compared to the inhibitor molecules alone.

Initiation of ventilation in extremely low birth weight infants often requires pressures greater than 30 cmH_2_O probably because endogenous surfactant concentrations and volume are low, and fetal lung fluid volumes are high as a result of immature fluid clearance pathways (Jobe and Ikegami, 1998a, b). To assess if CS could decrease opening airway pressure in the clinical scenario, compliance and tidal volume were measured after CS treatment in our preterm rabbit model which resulted in lungs that had increased compliance and higher tidal volume.

Mechanical injury triggers activation of the proinflammatory cytokine cascade leading to biotrauma. Even brief periods of IMV with supplemental O_2_ are enough to invoke proinflammatory cytokines. This cascade may promote injury in remote regions of the lung not exposed to significant mechanical insult. We tested the expression of inflammatory markers and found that TGFβ, Ang2, IL1β, and IL6 did increase following ventilation and hyperoxia exposure and decreased significantly after CS treatment. CHOP siRNA treatment increased the levels of IL1β and IL6. In contrast, Ang2 siRNA or miR34a Inh treatment alone or in combination with CS, led to a significantly decreased expression of the above two cytokines. There could be two possibilities of the findings in the CHOP siRNA group: IMV causes alveolar stretch which activates the integrated stretch response (ISR) pathway of which CHOP is a key player (Dolinay et al., 2017). It could be possible that the expression of CHOP could have been much higher after IMV due to which the dosing of CHOP siRNA was not enough to suppress CHOP following its activation by IMV, either alone or when given in combination with CS. As siRNA mediated delivery relies on charge complementation and complex formation between the vehicle and the test compound (Thomas et al., 2007), it could also be possible that the complex between CS and CHOP siRNA was not stable enough to allow for absorption in the alveolar epithelium. As a result, protein extravasation into the alveoli could have occurred, which further inhibited surfactant function and increased the infiltration of inflammatory cells, especially neutrophils (Carvalho et al., 2013). This could potentially be overcome with higher CHOP siRNA dosing, but additional studies are required.

Ventilation and hyperoxia triggered inflammation showing an increase in IL1β and IL6. Other than the Ang2 group, CS treatment did show a decrease in the expression of these two cytokines in the CHOP group and the miR34a Inh group; paradoxically when CS was dosed with CHOP siRNA, inflammation was still persistent in the CHOP group but not in the Ang2 siRNA group or miR34a Inh group. The most promising results were found in the miR34a Inh group when CS was given as an adjuvant. The discrepancy and variability of CS induced inflammatory response in the three experimental groups could be linked to tidal volume. Basing on the information that stretching causes surfactant release through calcium-mediated ion channels, it is reasonable to assume that during ventilation, large aggregates of surfactant are converted to inactive small aggregates which accumulate during the process and may inhibit the recovery response (Veldhuizen et al., 2001).

There were marked histopathological alveolar changes following IMV and hyperoxia exposure characterized by interstitial thickening, hyaline membrane formation and proteinaceous deposits. Hyaline membrane deposits were more pronounced even in the non-ventilated controls, which decreased after CS administration. Atelectasis was reversed after CS administration. In the Ang2 siRNA treated group CS did not make a significant difference to the hyaline membrane deposits. The scoring index was maximum in the CS treated animals compared to the rest of the groups in this experiment. Surprisingly, in the miR34a Inh group, the scoring index was highest in the non-ventilated controls as compared to ventilated counterparts probably because the lungs of kits receiving CS at birth inflated uniformly with constant distending pressure while the lungs of kits treated after a period of ventilation had a mix of aerated, partially aerated and atelectatic areas. This clearly shows that premature birth either naturally or by C-section can cause injury to the lungs as a result of the combinatorial effects of IMV and hyperoxia-induced inflammation, vascular injury, increased cell death and abnormal lung function.

In the present study, we found that in preterm kits, the vessels were thin walled, scantily distributed and disrupted in their alignment with the alveolar cells (dysmorphic); vWF staining was intense in all the ventilated groups, irrespective of CS treatment. Additionally, treatment with CS did improve the vascular remodeling as was evident from thicker pulmonary vessels and increased vWF staining as compared to unventilated controls. Inhibitors alone (either CHOP siRNA or Ang2 siRNA or miR34a Inh) also had a similar effect as that of CS, but a combination of CS + the small molecule inhibitors increased vessel density, vessel surface area, and improved the vascular remodeling. It is known that endothelial injury results in increased vWF staining (Patrick et al., 1993). Severe respiratory failure is characterized by diffuse pulmonary microvascular injury and produces marked increases in Factor VIII and vWF antigen in adults (Carvalho et al., 1982) as well as in premature newborns (Patrick et al., 1993).

Although cell death was more pronounced in the ventilated group as compared to non-ventilated controls, there was no difference in the rest of the groups following CS treatment. A similar observation has also been previously reported (May et al., 2004). Mokres et al. have reported that prolonged cyclic stretch of the developing lung or in adult rat type II lung epithelial cells in the absence of hyperoxia, can still cause apoptosis without a compensatory increase in cell proliferation (Hammerschmidt et al., 2004; Mokres et al., 2010).

With the standardized use of surfactant in neonatal intensive care units (NICUs), there is a need to understand the mechanism of action of this compound in combination with possible therapeutic drugs. Surfactant therapy may offer hope as an adjuvant therapy, along with small molecule inhibitors, as is evident from this study. Although there was some variability in a single experimental group, our study has undoubtedly opened the possibilities of adjuvant surfactant therapy with inhibitors of inflammation to suppress injury during IMV and hyperoxia exposure. In this study, of the three inhibitors used, miR34a inhibitor seemed to be the most promising compound to combat IMV and hyperoxia-induced lung injury in preterm rabbits.

## Data Availability Statement

All datasets generated for this study are included in the article/Supplementary Material.

## Ethics Statement

The animal study was reviewed and approved by Drexel University, Philadelphia, PA, United States, while the rabbit studies were reviewed and approved by Karolinska Instituet, Stockholm, Sweden.

## Author Contributions

PD, TC, FS, CC, NP, and VB contributed to the concept and design. PD, TC, BA, VP, JR, SB, and MS contributed to the acquisition of data. PD, TC, BA, FS, CC, NP, and VB contributed to the data analysis and interpretation. PD, TC, BA, FS, CC, and VB contributed to the drafting, editing and the critical revision for intellectual content. All authors have approved the final version of the submitted manuscript.

## Conflict of Interest

BA was employed by company GenomeRx US LLC, and FS, CC, and NP were employed by Chiesi Farmaceutici S.p.A. The remaining authors declare that the research was conducted in the absence of any commercial or financial relationships that could be construed as a potential conflict of interest.
