# Supplementary figures and images for: Small Molecule Inhibitor Adjuvant Surfactant Therapy Attenuates Ventilator- and Hyperoxia-Induced Lung Injury in Preterm Rabbits
Source: Front Physiol. 2020 Apr 9;11:266. doi: 10.3389/fphys.2020.00266 (PMC7160647; doi:10.3389/fphys.2020.00266)

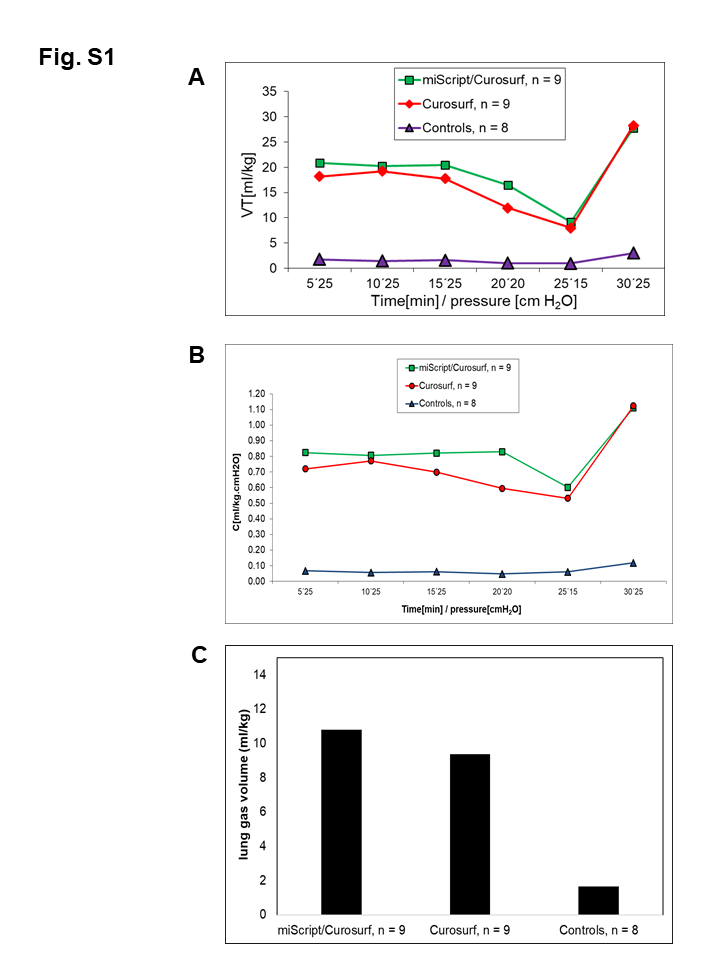

Supplement: Supplementary file 1 [file Image_1.TIF]

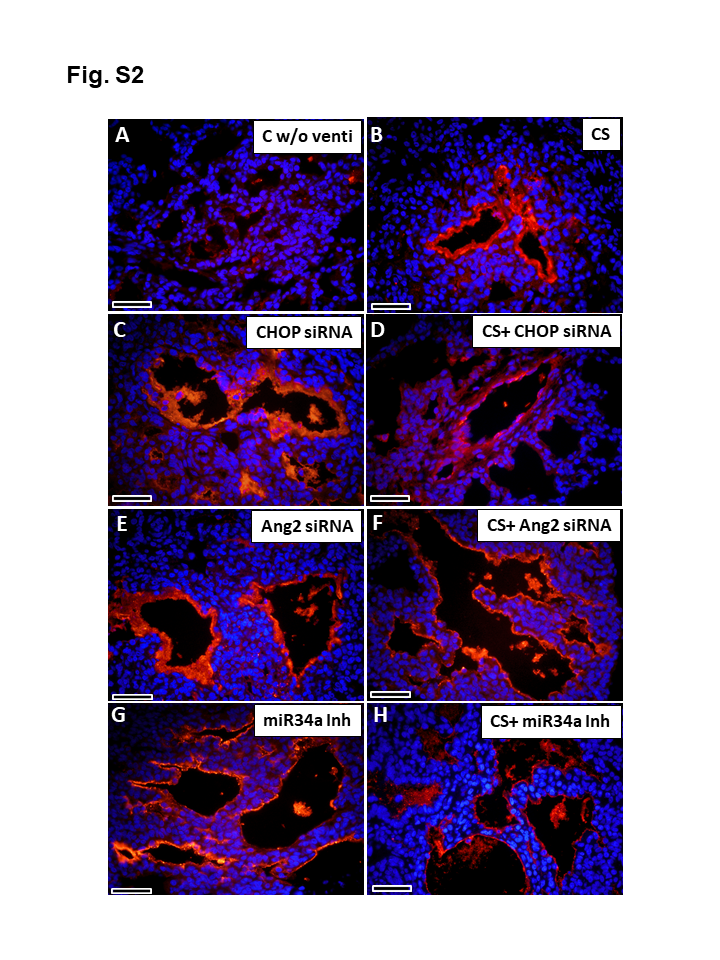

Supplement: Supplementary file 2 [file Image_2.TIF]
