# Supplementary material for: Small Molecule Inhibitor Adjuvant Surfactant Therapy Attenuates Ventilator- and Hyperoxia-Induced Lung Injury in Preterm Rabbits
Source: Front Physiol. 2020 Apr 9;11:266. doi: 10.3389/fphys.2020.00266 (PMC7160647; doi:10.3389/fphys.2020.00266)
Supplement: Supplementary file 3 [file Data_Sheet_1.pdf]

Fig. S1: Pulmonary Function in G27 rabbit kits. (A) Tidal volume in preterm rabbits of 27 days gestational age ventilated with 100% oxygen and standardized pressures without PEEP. **(B)** Compliance in preterm rabbits of 27 days gestational age ventilated with 100% oxygen and constant pressures without PEEP. **(C)** Lung gas volumes in rabbits of 27 days gestational age ventilated with 100% oxygen and constant pressures without PEEP. The same set of rabbits were used for experiments (**A**), (**B**) and (**C**). Addition of miR34a Inh to Curosurf had no negative effect on tidal volume, compliance and lung gas volume.

Fig. S2. Immunostaining of pulmonary vessels after CS treatment. Representative images of pulmonary artery and pulmonary vessels showing expression of vWF after adjuvant treatment with CS and 3 small molecule inhibitors. Unventilated controls born prematurely have fewer blood vessels with a thin endothelial lining (**A**) while in CS controls, the staining of pulmonary arterial wall is more regular (**B**). CHOP siRNA was not able to improve the vascular pattern (**C**); however, a combination of CHOP siRNA + CS, was able to improve vascular remodeling (**D**). Ang2 siRNA showed a slight improvement (**E**) as compared to CHOP siRNA (**C**). When given adjuvantly with CS, the endothelial lining was uniform, complete and continuous (**F**). miR34a Inh by itself (**G**) or when used in combination with CS (**H**) also showed a uniform and continuous endothelial lining. Scale bars: 100µm; Red: vWF staining; Blue: DAPI nuclear staining.

Supplemental Table 1: Number of preterm rabbits at G28 surviving different time periods. Both control animals and Curosurf treated animals were ventilated for 4 h with 100% oxygen, a constant tidal volume of 6 – 7 ml/kg and a PEEP of 3 cmH_2O

**Maximum Survival time (min)**	**Controls**	**CS**
<180	1	1
180	2	0
240	8	6

Supplemental Table 2: Primer sequences

**Gene**	**Forward primer (5’ to 3’)**	**Reverse primer (5’ to 3’)**
**TGFβ1**	5’ AAGGGCTACCACGCCAACTT 3’	5’ CCGGGTTGTGCTGGTTGTAC 3’
**Ang1**	5’ TGTGCCCTCATGCTTACAGG 3’	5’ CGTTCAGTTTGCCGTGGTTT 3’
**Ang2**	5’ AGGAGGCTGGTGGTTTGATG 3’	5’ TTGTGGCCTTGAGCGAGTAG 3’

**CHOP**	5’ TTGCCTTTCTCCTTCGGGAC 3’	5’ TCCAGGGGGTGAGACATAGG 3’
**IL1β**	5' GCCGATGGTCCCAATTACAT 3'	5' ACAAGACCTGCCGGAAGCT 3'
**IL6**	5' GAAAACACCAGGGTCAGCAT 3'	5' CAGCCACTGGTTTTTCTGCT 3'
**GAPDH**	5' GAATCCACTGGCGTCTTCAC 3'	5' CGTTGCTGACAATCTTGAGAGA 3'

Supplemental Table 3: Number of preterm rabbits at G-28 with different survival times. Control animals were sacrificed before the first breath and are not included in the table.

	**Experiment 1**			**Experiment 2**			**Experiment 3**		
**Maximum survival time(min)**	**CS**	**CHOP siRNA + CS**	**Ddit3**	**CS**	**Ang2 siRNA + CS**	**Ang2siRNA**	**CS**	**miR34a Inh + CS**	**miR34a Inh**
<90	1	5	1	2	2	1	0	0	1
90 - 179	0	7	3	3	1	3	2	2	0
180-239	1	2	5	0	0	1	1	0	1
240	11	2	3	5	5	4	6	6	5
